# Induction of Live Cell Phagocytosis by a Specific Combination of Inflammatory Stimuli

**DOI:** 10.1016/j.ebiom.2017.07.011

**Published:** 2017-07-13

**Authors:** Takamasa Ishidome, Takeshi Yoshida, Rikinari Hanayama

**Affiliations:** a Department of Immunology, Kanazawa University Graduate School of Medical Sciences, 13-1 Takara, Kanazawa, Ishikawa 920-8640, Japan; b Laboratory of Immune Network, WPI Immunology Frontier Research Center (IFReC), Osaka University, 3-1 Yamada-oka, Suita, Osaka 565-0871, Japan; c PRESTO, Japan Science and Technology Agency (JST), 4-1-8 Honcho, Kawaguchi, Saitama 332-0012, Japan

**Keywords:** Phagocytosis, Hemophagocytosis, Cell-in-cell structures, Cytokine storm, Inflammation, Macrophage activation syndrome

## Abstract

Conditions of severe hyper-inflammation can lead to uncontrolled activation of macrophages, and the ensuing phagocytosis of live cells. However, relationships between inflammatory stimuli and uncontrolled phagocytosis of live cells by macrophages are poorly understood. To identify mediators of this process, we established phagocytosis assays of live cells by stimulating macrophages with CpG DNA, interferon-γ, and anti-interleukin-10 receptor antibody. In this model, various cell surface receptors were upregulated on macrophages, and phagocytosis of live cells was induced in a Rac1-dependent manner. Subsequent inhibition of the ICAM-1, VCAM-1, and both of these receptors abolished *in vitro* and *in vivo* phagocytosis of live T cells, myeloid cells, and B cells, respectively. Specifically, the reduction in lymphocyte numbers due to *in vivo* activation of macrophages was ameliorated in *Icam-1*-deficient mice. In addition, overexpression of ICAM-1 or VCAM-1 in non-phagocytic NIH3T3 cells led to active phagocytosis of live cells. These data indicate molecular mechanisms underlying live cell phagocytosis induced by hyper-inflammation, and this experimental model will be useful to clarify the pathophysiological mechanisms of hemophagocytosis and to indicate therapeutic targets.

## Highlights

• A specific combination of inflammatory stimuli induce phagocytosis of live cells
• ICAM-1 and VCAM-1 induced on macrophages mediate live cell phagocytosis
• Hemophagocytosis was ameliorated in *Icam-1*-deficient mice

Under severe inflammatory conditions, macrophages are hyper-activated and start to eat live blood cells and their precursors, a process known as “hemophagocytosis”. Hemophagocytosis accompanies with inflammatory diseases such as infections, malignancies, or autoimmune diseases, but molecular mechanisms that cause hemophagocytosis remain unclear. We established *in vitro* cell culture model of hemophagocytosis and found that ICAM-1 and VCAM-1 were induced on macrophages by a specific combination of inflammatory stimuli to mediate hemophagocytosis. We expect that our findings will facilitate the development of therapeutic approaches against the hemophagocytic syndromes.

## 1 Introduction

In our body, billions of cells perform apoptotic cell death every day and are removed by phagocytes such as macrophages. This process is known as efferocytosis and is dependent on recognition and selective phagocytosis of dead cells by phagocytes (Nagata et al., 2010). Among plasma membrane molecules, the phospholipid phosphatidylserine is important for phagocyte recognition of cells to engulf (Segawa and Nagata, 2015). Hence, in live cells, phosphatidylserine is exclusively maintained in the inner leaflet of the lipid bilayer through the actions of phospholipid flippases (Segawa et al., 2014). During apoptosis, phospholipid flippases are inactivated and phospholipid scramblases are activated, leading to exposure of phosphatidylserine to the outer leaflet (Suzuki et al., 2013). Subsequently, phagocytic recognition and signaling is mediated by phosphatidylserine-binding proteins such as MFG-E8, Tim4, BAI1, and Gas6 (Nagata et al., 2010). In addition, cell surface expression of CD31 or CD47 on live cells is protective against phagocytosis, and these molecules are downregulated during apoptosis (Brown et al., 2002, Gardai et al., 2005). Together these mechanisms prevent phagocytosis of live cells and ensure phagocytosis of apoptotic cells.

In contrast, phagocytosis of live cells can be induced by uncontrolled activation of macrophages by strong immunologic conditions such as systemic infection, autoimmunity, and malignancy. Specifically, phagocytosis of live blood cells and their precursors by macrophages is known as hemophagocytosis. It is often observed in hemophagocytic lymphohistiocytosis (HLH) and macrophage activation syndrome (MAS), which are characterized by overwhelming immune activation and excessive production of inflammatory cytokines (Janka, 2012, Grom et al., 2016). Several studies have defined hemophagocytosis as a type of efferocytosis. For example, CD47 expression was reportedly downregulated in live hematopoietic stem cells from HLH patients (Kuriyama et al., 2012). Moreover, numbers of apoptotic erythrocytes were increased in a mouse model of HLH (Ohyagi et al., 2013). However, because abnormal activation of macrophages by inflammatory cytokines such as interferon-γ (IFN-γ) is the primary cause of hemophagocytosis (Zoller et al., 2011), these observations offer little to the understanding of pathogenic mechanisms. In particular, it remains unclear how inflammatory cytokines induce macrophages to engulf live cells.

We therefore considered that establishment of an *in vitro* cell culture model of live cell phagocytosis will be useful not only for identifying phagocytic receptors of live cells but also for clarifying the pathogenesis of hemophagocytosis to indicate therapeutic targets. However, specific *in vitro* stimuli inducing phagocytosis of live cells by macrophages remain elusive. Recently, repeated injections of Toll-like receptor (TLR) 9 ligand, CpG DNA into mice have been reported to induce MAS-like diseases and hemophagocytosis (Behrens et al., 2011). Accordingly, we tried to induce phagocytosis of live cells in cultured macrophages using CpG DNA treatments.

## 2 Materials and Methods

### 2.1 Mice, Cells, and Reagents

C57BL/6 mice were purchased from SLC, Japan and C57BL/6-Tg (CAG-EGFP) mice (Okabe et al., 1997) were a kind gift from M. Okabe. *Icam*-1 KO mice were generated by pronuclear injection of CRISPR/Cas9 pX330 vectors targeting mouse *Icam-1* exon-4 (CGCTGCGTTTTGGAGCTAGCGG) and exon-6 (TCCTAAGATGACCTGCAGACGG) to introduce frameshift mutations (PAM sequences are underlined). All mice were housed in a pathogen-free facility and all animal experiments were performed according to protocols that were approved by the Animal Research Committee of Kanazawa University, Japan. Bone marrow-derived macrophages (BMDMs) were generated by culturing bone marrow cells from femurs and tibias of mice for 4–6 days in high glucose DMEM (Nacalai, Japan) supplemented with 10% FBS (Biowest), 1% penicillin/streptomycin, and 10 units/ml of macrophage colony-stimulating factor (M-CSF), which was prepared using conditioned medium from human M-CSF overexpressing mouse L929 cells (Takeshita et al., 2000). Primary cultures of thioglycollate-elicited peritoneal macrophages (pMACs) and bone marrow-derived dendritic cells (BMDCs) were prepared as described previously (Hanayama et al., 2002, Miyasaka et al., 2004). All cell lines were obtained from RIKEN Bio Resource Center (Japan) and tested for mycoplasma contamination. Cells were treated either with recombinant IFN-γ, the Rac1 inhibitor NSC23766 (Wako, Japan), cycloheximide (Nacalai, Japan), CpG ODN-1826 (5′- TCCATG ACGTTCCTGACGTT-3′, Hokkaido System Science, Japan), or BAPTA-AM (Dojindo, Japan). Monoclonal antibodies against mouse B220 (RA3-6B2, RRID:AB_312996), CD3 (17A2, RRID:AB_312661), CD4 (GK1.5, RRID:AB_312696), CD8a (53-6.7, RRID:AB_312751), CD11b (M1/70, RRID:AB_312794), CD68 (FA11, RRID:AB_10575475), Gr-1 (RB6-8C5, RRID:AB_313368), ICAM-1 (YN1/1.7.4, RRID:AB_313700), IL-10R (1B1.3a, RRID:AB_313521), Integrin α_V_ (RMV-7, RRID:AB_2265155), Integrin β_3_ (2C9.G2, RRID:AB_313086), LAMP-1 (1D4B, RRID:AB_572003), LFA-1α (M17/4, RRID:AB_10694867), PECAM-1 (MEC13.3, RRID:AB_312918), VCAM-1 (429, RRID:AB_313209), VLA-4α (9C10, RRID:AB_2129608), and VLA-5α (5H10-27, RRID:AB_313065), and rat IgG1κ (RTK2071, RRID:AB_326519), IgG2a κ (RTK2758), IgG2b κ (RTK4530, RRID:AB_2086803), and Armenian hamster IgG (HTK888) isotype control antibodies, and mouse IL-10 ELISA Kit were purchased from BioLegend. The D89E mutant of mouse MFG-E8 was prepared as described previously (Hanayama et al., 2002).

### 2.2 Plasmids and Transfection

DNA fragments for full length coding sequences of mouse *Icam-1* and *Vcam-1* were prepared using RT-PCR after RNA extraction from CpG, IFN-γ, and αIL-10 cotreated BMDMs using the following primers: ICAM-1-Fw, 5′-CCCGGATCCCTACCATGGCTTCAACCCGT-3′ and ICAM-1-Rv, 5′-AAAGCGGCCGCTCAGGGAGGTGGGGC-3′ (*Bam*HI and *Not*I sites are underlined), and VCAM-1-Fw, 5′-ATTAATTAAGCCACCATGCCTGTGAAGAGGTC-3′ and VCAM-1-Rv, 5′-AAAGCGGCCGCCTACACTTTGGATTTCTGTGC -3′ (*Pac*I and *Not*I sites are underlined). PCR fragments were digested using *Bam*HI/*Pac*I and *Not*I restriction endonucleases, and were inserted into pMXs retrovirus vectors (Kitamura et al., 2003). The pMXs-Rac1 construct was a gift from S. Nagata. Retroviral plasmid lipofection into PLAT-E packaging cells (Morita et al., 2000) was performed using FuGENE6 (Promega), and 48 h later, NIH3T3 cells or BMDMs were infected with culture supernatants in the presence of 10 μg/ml polybrene to establish stable transformants. NIH3T3 transformants expressing integrin α_V_β_3_ were generated as described previously (Hanayama et al., 2002).

### 2.3 *In Vitro* Phagocytosis Assays

Phagocytes (1 × 10^5^ cells) were cultured on 24-well plates (Corning) for flow cytometric analyses or on NUNC Lab-Tek II 8-well chamber glass slides (Thermofisher) for microscope analyses, and were then activated using various combinations of IFN-γ (100 U/ml), CpG ODN-1826 (0.5 μg/ml), and/or αIL-10R (1.25 μg/ml) in the absence or presence of cycloheximide (1 μg/ml) or the Rac1 inhibitor NSC23766 at 50, 100, or 200 μM for 20 h. Prey cells such as thymocytes, splenocytes, and myeloid cells were freshly prepared from 4 to 6 week old C57BL/6 mice. Myeloid cells were prepared from bone marrow by depleting T and B cells using a FACSAria cell sorter (BD Biosciences) with αCD3 and αB220 antibodies. Apoptosis was induced in thymocytes using 10 μM dexamethasone treatments for 4 h, and in myeloid cells by UV irradiation at 200 J/cm^2^ and incubation for 2 h. For flow cytometric analyses, prey cells were washed twice with PBS and were incubated for 30 min with 1 μM CellTracker green dye (CMFDA) (Thermofisher). Reactions were stopped by adding 1 ml of FBS and the cells were then washed twice with DMEM containing 10% FBS. The labeled cells (1 × 10^6^ cells) were added to BMDMs that had been pre-treated with or without 4 μg/ml antibodies, 10 or 20 μM BAPTA-AM, or 7 μg/ml MFG-E8 D89E for 30 min. Phagocytosis proceeded for 2.5 h at 37 °C and prey cells that remained free were removed by washing twice with PBS. Phagocytes were collected from the plates by trypsinization, and were washed and suspended in PBS containing 2% FBS and 0.02% NaN3. Percentage phagocytosis was determined in triplicates by quantifying the percentage of CMFDA-positive phagocytes using FACSVerse (BD Biosciences). For microscope analyses, prey cells (1 × 10^6^ cells) were added to GFP-labeled phagocytes on glass slides that had been pre-treated with or without antibodies at 4 (for BMDMs) or 10 μg/ml (for NIH3T3 cells, pMACs, or BMDCs) for 30 min. In some experiments, prey cells were pre-labeled with 0.1 μg/ml pHrodo Red (Thermofisher). After co-culturing for 2.5 h, cells were washed twice with PBS and fixed in 4% paraformaldehyde (PFA)/PBS. When co-cultured with splenocytes, the phagocytes were permeabilized with ice-cold acetone and were stained with APC αB220, αCD4, or αCD8a antibodies in PBS containing 1% BSA to identify cell types of the engulfed cells. The cells were then mounted with ProLong Gold Antifade (Thermofisher) containing 1% DAPI (Dojindo, Japan), and were observed using an FV10i confocal microscope (Olympus, Japan). Engulfed cells can be detected as dark spots in the cytoplasm and total numbers of engulfed cells of 200 phagocytes (50 cells/field × 4 fields) were counted blindly, and phagocytosis indexes were determined as average numbers of engulfed cells per 100 phagocytes.

### 2.4 Detection of Live and Apoptotic Cells

TUNEL, JC-1, or Annexin V staining was performed using ApopTag Fluorescein Direct *In Situ* Apoptosis Detection Kits (Merck Millipore), MitoPT JC-1 Assay Kit (ImmunoChemistry Technologies), or FITC-Annexin V (BioLegend), respectively. For electron microscope analyses, BMDMs were cultured on Cell Desk (Sumitomo Bakelite, Japan) polystyrene cover slips. After phagocytosis, cells were fixed using 2% formaldehyde and 2.5% glutaraldehyde in 0.1 M sodium-phosphate buffer (pH 7.4) and were washed three times for 5 min in the same buffer. Cells were then post-fixed for 1 h in 0.1 M sodium-phosphate buffer (pH 7.4) containing 1% osmium tetroxide and 1% potassium ferrocyanide, and were then dehydrated in a graded series of ethanol and embedded in Epon812 (TAAB, UK). Subsequently, 80 nm ultra-thin sections were stained with a saturated solution of uranyl acetate and lead citrate. Electron micrographs were obtained using a JEM-1011 transmission electron microscope (JEOL, Japan).

### 2.5 Microarray and qPCR Analyses

BMDMs were stimulated with CpG (0.063 μg/ml), IFN-γ (100 U/ml) + αIL-10R (1.25 μg/ml), or with all three agents at 37 °C for 20 h. Total RNA from each BMDM sample was then extracted using RNeasy Plus Mini Kits (QIAGEN) according to the manufacturer's instructions. Microarray analyses were performed by MBL, Japan, using SurePrint G3 Mouse Gene Expression 8 × 60 K Microarrays (Agilent). For qPCR analyses, RNA was reverse transcribed using ReverTra Ace qPCR master mix (TOYOBO, Japan), and cDNA products were amplified using a LightCycler 96 (Roche) with Universal SYBR Select master mix (Thermofisher). Data were analyzed using the delta Ct method and were normalized to *β-actin* RNA expression in each sample. The original microarray data were deposited in GEO database under accession number GSE90881. The primers for real-time PCR were as follows: β-actin-Fw, 5′-CTTTGCAGCTCCTTCGTTG-3′ and β-actin-Rv, 5′-CGATGGAGGGGAATACAGC-3′; ICAM-1-Fw, 5′-GTCCGCTGTGCTTTGAGAAC-3′ and ICAM-1-Rv, 5′-TGAGGTCCTTGCCTACTTGC-3′; VCAM-1-Fw, 5′-GAAATGCCACCCTCACCTTA-3′ and VCAM-1-Rv, 5′-CGGGGGAGATGTCAACAATA-3′; LFA-1α-Fw, 5′-CAGAACAAGAACCCCGATGT-3′ and LFA-1α-Rv, 5′-CCTGGCACCAGACTCTTCTT-3′; and VLA-4α-Fw, 5′-AATGCCTCAGTGGTCAATCC-3′ and VLA-4α-Rv, 5′- TCTCCTCCAGGCATGTCTTC-3′.

### 2.6 *In Vivo* Phagocytosis Assays

*E. coli* DH5α (Toyobo, Japan) cells were grown at 37 °C in 100 ml of LB medium until the mid-exponential phase (OD_600_ = 0.8–1.0). Cells were then collected by centrifugation at 4000 ×* g* for 10 min and were inactivated by mixing with 50 ml of 70% (v/v) ethanol for 30 min. Mixtures were then centrifuged and cells were washed and suspended in PBS, and were then treated with UV irradiation for 30 min. Subsequently, 200 μg aliquots of dead *E. coli* were injected into peritoneal cavities of 8–10 week old male mice, and after 2 days, 4 × 10^7^ CMFDA-labeled freshly-isolated live cells were injected into peritoneal cavities with blocking antibodies or corresponding isotype controls (10 μg). After 2.5 h, peritoneal cells were collected and plated on glass slides for 1 h to allow attachment of macrophages. Attached macrophages were then fixed and stained with antibodies against CD11b and Gr-1, and were observed using a confocal microscope. Total numbers of engulfed cells in 50 CD11b^+^/Gr-1^−^ peritoneal macrophages (10 cells/field × 5 fields) were counted, and phagocytosis indexes were determined as the number of engulfed cells per 100 macrophages. To induce *in vivo* hemophagocytosis and a peripheral pancytopenia, male mice were injected i.p. on days 0, 2, 4, 6, and 8 with PBS or dead *E. coli* (20 mg). On day 7, peripheral blood was sampled by cheek bleed, and blood cell counting was performed with FACSCanto II (BD Biosciences). The number of T or B cells was counted by staining with αCD3 or αB220 antibody after RBC lysis. On day 10, mice were euthanized, and spleens were taken for immunohistochemical analysis with antibody against CD68.

## 3 Results

### 3.1 Combinations of Inflammatory Stimuli Induce Phagocytosis of Live Cells

**Fig. 1:**
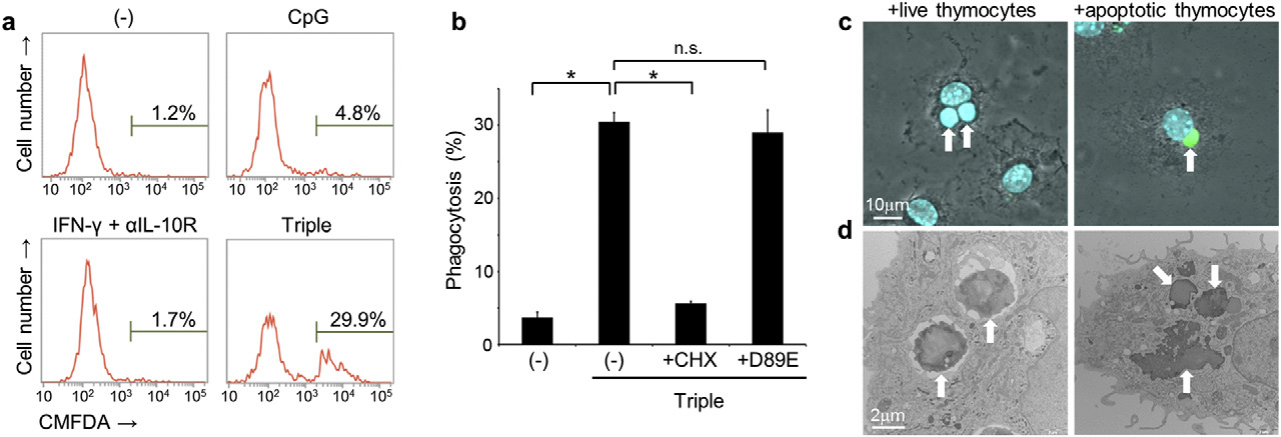
Induction of live cell phagocytosis in cell cultures. (a) BMDMs were stimulated or not (−) with CpG DNA (0.5 μg/ml), IFN-γ (100 U/ml) and αIL-10R (1.25 μg/ml), or with CpG, IFN-γ, and αIL-10R (Triple) for 20 h, and were then co-cultured with CellTracker green dye (CMFDA)-labeled freshly-isolated live thymocytes for 2.5 h. Percentage of macrophages that contained CMFDA-positive live thymocytes were then analyzed using flow cytometry. (b) BMDMs were stimulated or not using cotreatment with CpG, IFN-γ, and αIL-10R (Triple) before co-culturing with CMFDA-labeled live thymocytes, and percentages of macrophages that performed phagocytosis were then analyzed using flow cytometry. BMDMs were also treated with or without (−) cycloheximide (CHX, 1 μg/ml) throughout the cotreatment period, or the D89E mutant of MFG-E8 (7 μg/ml) 30 min before incubation with thymocytes; *p < 0.01; n.s., not significant, ANOVA followed by Tukey *post hoc* test. (c and d) BMDMs activated with the combination of CpG, IFN-γ, and αIL-10R, were incubated with live or apoptotic thymocytes, and then analyzed using TUNEL (green) and DAPI (blue) staining (c) or electron microscopy (d). Arrows indicate thymocytes that were engulfed by macrophages. (For interpretation of the references to colour in this figure legend, the reader is referred to the web version of this article.)

To reconstitute phagocytosis of live cells in cell culture, BMDMs were co-cultured with CMFDA-labeled freshly-isolated live thymocytes for 2.5 h and percentages of macrophages containing live thymocytes were quantified using flow cytometry. Unstimulated macrophages did not engulf live cells, whereas 4.8% of macrophages engulfed live cells following 20 h treatments with CpG DNA (Fig. 1a). Although subsequent experiments with IFN-γ and anti-IL-10 receptor antibody (αIL-10R) showed no induction of live cell phagocytosis, combined treatments with CpG, IFN-γ, and αIL-10R led to efficient live cell phagocytosis in 29.9% of macrophages. This synergistic effect was completely abolished by the presence of the protein synthesis inhibitor cycloheximide, indicating that induction of live cell phagocytosis by CpG, IFN-γ, and αIL-10R requires synthesis of certain proteins (Fig. 1b). During incubation with macrophages for 2.5 h, a few thymocytes spontaneously undergo apoptosis, and about 10% of the cells became Annexin V-positive (Fig. S1a). To determine whether the macrophages only engulfed live cells, experiments were performed in the presence of milk fat globule-EGF factor 8 protein (MFG-E8) carrying a D89E substitution, which blocks phagocytosis of apoptotic cells (Hanayama et al., 2002), but it did not inhibit the phagocytosis (Fig. 1b). TUNEL staining of dead cell nuclei confirmed the absence of apoptotic cells in macrophages which had been co-cultured with live cells (Fig. 1c). The engulfed live cells were also confirmed for maintaining mitochondrial membrane potential, which was lost in the control apoptotic cells (Fig. S1b). In further experiments, we analyzed cell morphologies of engulfed cells using transmission electron microscopy, and found the presence of live cells with intact plasma membranes in macrophage phagosomes (Fig. 1d). In contrast, the control apoptotic cells were degraded and only condensed nuclei remained inside the macrophages.

### 3.2 Distinct Post-phagocytic Responses After Phagocytosis of Live and Apoptotic Cells

**Fig. 2:**
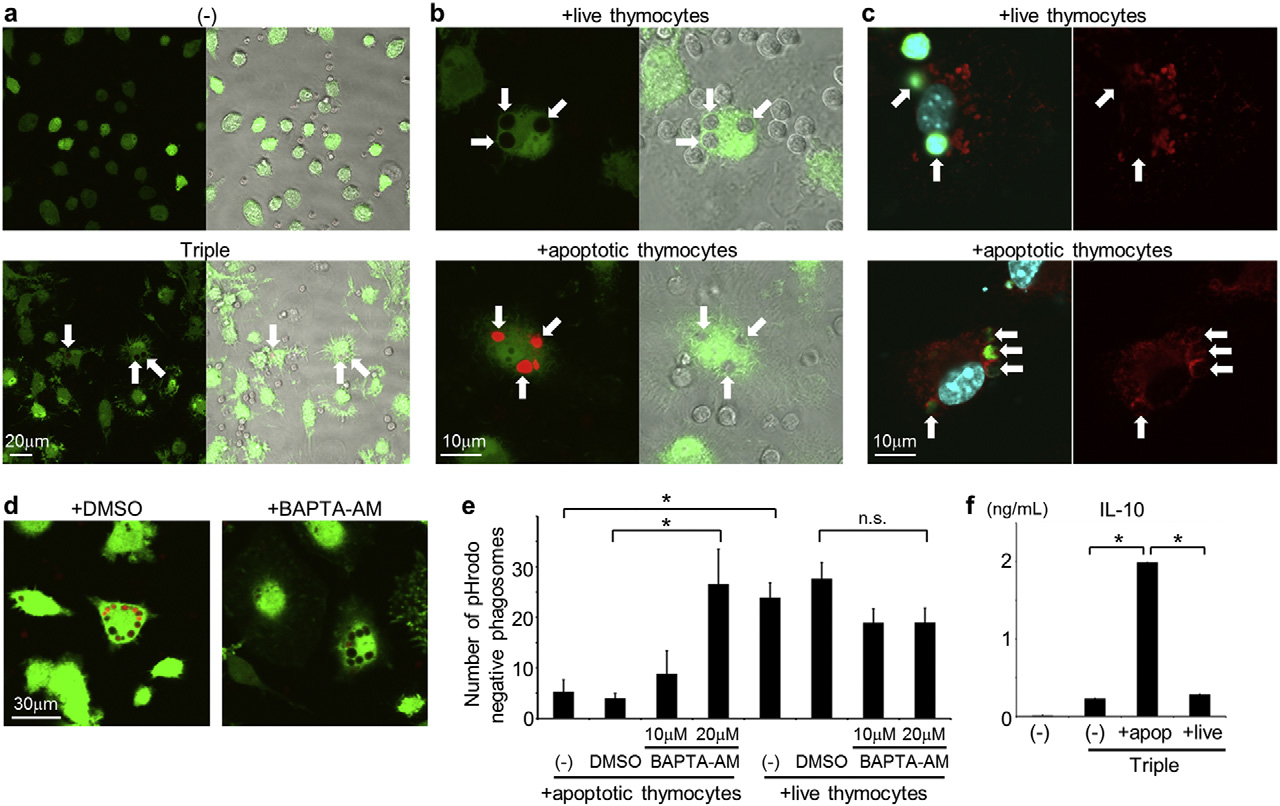
Delayed phagosome acidification after phagocytosis of live cells. (a) BMDMs were established from GFP-transgenic mice (GFP-BMDMs) and were treated or not (−) with CpG, IFN-γ, and αIL-10R in combination (Triple). After co-culture with pH-rodo-labeled live thymocytes, cells were observed using confocal microscopy (left panels) and staining profiles were merged with the phase contrast images in the right panels. The pH-rodo dye emits a red signal in acidic environments; Arrows indicate engulfed live thymocytes. (b) GFP-BMDMs were activated by cotreatment with CpG, IFN-γ, and αIL-10R and were co-cultured with pH-rodo-labeled live or apoptotic thymocytes for confocal microscope analyses (left panels). Staining profiles were merged with the phase contrast images in the right panels. Arrows indicate engulfed thymocytes. (c) BMDMs containing CMFDA-labeled live or apoptotic thymocytes (green) were stained with antibody against LAMP-1 (red). Cells were counter-stained with DAPI (blue) and were then observed using confocal microscopy (left panels). Images of LAMP-1 staining only are shown in the right panels. Arrows indicate engulfed thymocytes. (d and e) GFP-BMDMs stimulated with CpG, IFN-γ, and αIL-10R in combination were pre-treated with or without (−) DMSO or BAPTA-AM (20 μM) for 30 min. Macrophages were then co-cultured with pHrodo-labeled apoptotic thymocytes and were observed using a confocal microscope (d). Numbers of the pHrodo-negative phagosomes per 100 macrophages after co-culture with pHrodo-labeled live or apoptotic thymocytes were determined and average values were plotted with standard deviations (s.d.) (e); *p < 0.01; n.s., not significant, ANOVA followed by Tukey *post hoc* test. (f) BMDMs were activated or not with CpG, IFN-γ, and αIL-10R (Triple) and were then co-cultured with or without (−) apoptotic (apop) or live thymocytes. IL-10 protein levels in culture supernatants were measured by ELISA; *p < 0.01, ANOVA followed by Tukey *post hoc* test. (For interpretation of the references to colour in this figure legend, the reader is referred to the web version of this article.)

To identify mechanism that is associated with the presence of live cells inside phagosomes, we investigated phagosome maturation using pHrodo-labeled thymocytes, which emit red fluorescence following acidification due to phagosome–lysosome (P–L) fusion (Miksa et al., 2009). GFP-labeled macrophages were also used to detect engulfed cells as dark spots in the cytoplasm. These experiments confirmed that live cells were only internalized by macrophages following cotreatment with CpG, IFN-γ, and αIL-10R (Fig. 2a). Subsequent comparisons of phagosome acidification after phagocytosis of live and apoptotic cells, which were equally labeled with pHrodo dye (data not shown), showed no red signals in macrophages containing live cells but strong signals from those containing apoptotic cells (Fig. 2b). In agreement, staining with the lysosome marker αLAMP-1 showed that lysosomes only co-localized with phagosomes containing apoptotic cells (Fig. 2c). These results indicate that P–L fusion is promoted after phagocytosis of apoptotic cells but not after phagocytosis of live cells. The defect P–L fusion causes impaired degradation of engulfed cells, which might lead to persistence of the cells that can be detected in the bone marrow and spleen of HLH patients.

Previously, P–L fusion was associated with elevated cytosolic free calcium levels (Jaconi et al., 1990). Thus, to further define the present mechanisms, we performed experiments with the cell-permeable calcium chelator BAPTA-AM. Pre-treatment of macrophages with BAPTA-AM increased numbers of pHrodo-negative phagosomes after phagocytosis of apoptotic cells to levels that were observed following phagocytosis of live cells, which were not affected by the treatment (Fig. 2d and e). These data indicate that, unlike efferocytosis, live cell phagocytosis in this model is not accompanied by increased calcium signaling, suggesting that post-phagocytic responses may also differ. As previously reported (Voll et al., 1997), phagocytosis of apoptotic cells promoted the secretion of anti-inflammatory cytokines such as IL-10 from macrophages, whereas phagocytosis of live cells did not (Fig. 2f). Hence phagocytosis of live cells reconstituted in the present model likely did not induce immunosuppressive effects.

### 3.3 Stimulation With CpG, IFN-γ, and αIL-10R Induces ICAM-1 for Phagocytosis of Live T Cells

**Fig. 3:**
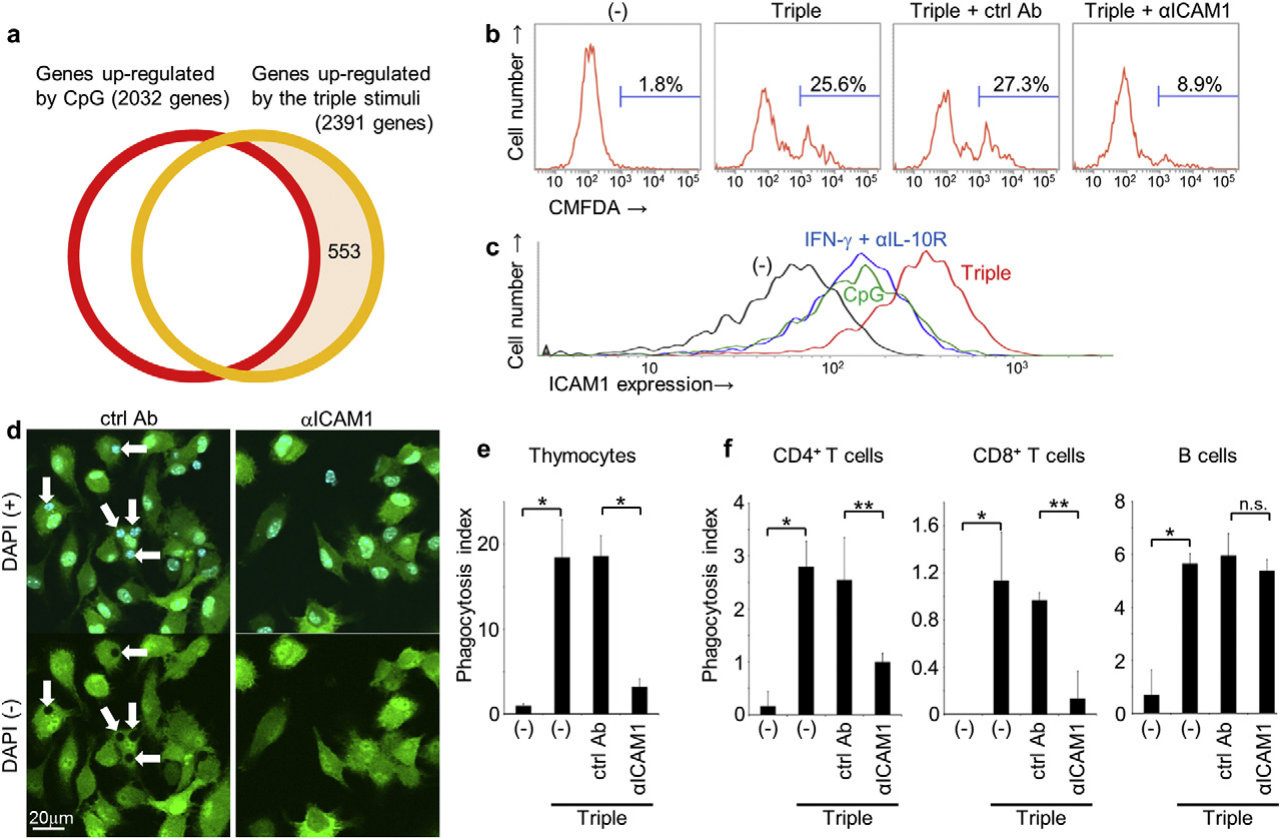
Up-regulation of ICAM-1 promotes phagocytosis of live T cells. (a) Gene expression levels in BMDMs were compared using DNA microarray analyses. Control BMDMs were treated with IFN-γ + αIL-10R and genes that were up-regulated > 2-fold following stimulation with CpG DNA (red circle) or CpG, IFN-γ, and αIL-10R (yellow circle) were identified. Genes that were up-regulated only by CpG, IFN-γ, and αIL-10R (553 genes) were selected for further analysis. (b) BMDMs were activated or not (−) using cotreatment with CpG, IFN-γ, and αIL-10R (Triple) and were co-cultured with CMFDA-labeled live thymocytes in the absence or presence of 4 μg/ml αICAM-1 antibody (αICAM-1) or its isotype control antibody (ctrl Ab). Percentages of CMFDA-positive macrophages were then quantified using flow cytometry. (c) BMDMs were stimulated with or without (−) indicated treatments for 20 h, and cell surface expression levels of ICAM-1 were analyzed using flow cytometry. (d) GFP-BMDMs were activated with CpG, IFN-γ, and αIL-10R cotreatment and were co-cultured with live thymocytes in the presence of αICAM-1 or isotype ctrl Ab. Cells were counter-stained with DAPI and were then observed using a confocal microscope (upper panels). Images without DAPI staining are shown in the lower panels to identify engulfed cells as dark spots in the cytoplasm. Arrows indicate engulfed thymocytes. (e) GFP-BMDMs were activated or not with CpG, IFN-γ, and αIL-10R cotreatment (Triple) and were co-cultured with live thymocytes in the absence (−) or presence of αICAM-1 or isotype ctrl Ab. Numbers of engulfed live thymocytes per 100 macrophages were determined as phagocytosis indexes, and average values were plotted with s.d.; *p < 0.01, Student's *t*-test. (f) GFP-BMDMs were activated or not using combined treatment with CpG, IFN-γ, and αIL-10R (Triple) and were co-cultured with live splenocytes in the absence (−) or presence of αICAM-1 or isotype ctrl Ab. Cells were fixed and permeabilized, and were then stained for CD4, CD8, or B220 for confocal microscope observations. Phagocytosis indexes of each engulfed cell type were determined; *p < 0.01, **p < 0.05; n.s., not significant, Student's *t*-test. (For interpretation of the references to colour in this figure legend, the reader is referred to the web version of this article.)

To identify mediators of live cell phagocytosis that are upregulated following cotreatment with CpG, IFN-γ, and αIL-10R, we examined gene expression profiles of BMDMs using microarrays of about 40,000 mouse genes (Fig. 3a). These experiments showed > 2-fold increases in the expression of 2391 genes in cells that were treated with CpG, IFN-γ, and αIL-10R, compared with those treated with IFN-γ + αIL-10R only, which did not induce live cell phagocytosis (Fig. 1a). In addition, 2032 genes were upregulated following stimulation with CpG alone, which also did not induce live cell phagocytosis, and comparisons with CpG, IFN-γ, and αIL-10R treated macrophages identified 553 specifically upregulated genes by the cotreatment. Among these upregulated genes, the cell surface proteins such as PECAM-1, ICAM-1, VCAM-1, and VLA-5 were selected as candidate receptors for live cells, and were targeted using specific monoclonal antibodies to inhibit live cell phagocytosis (Fig. S2a). In these experiments, αPECAM-1, αVCAM-1, and αVLA-5 did not inhibit the phagocytosis of live thymocytes. On the other hand, αICAM-1 antibody, but not the isotype control antibody, decreased the percentage of macrophages containing live thymocytes from 25.6% to 8.9% (Fig. 3b). ICAM-1 is a member of the immunoglobulin superfamily, which mediates cell–cell interactions and the ensuing signal transduction for T cell activation and leukocyte recruitment (Vestweber, 2015). Although cell surface expression of ICAM-1 was also weakly induced by CpG or IFN-γ + αIL-10R, its expression was 2.5-fold greater in the presence of CpG, IFN-γ, and αIL-10R (Fig. 3c). Microscope observations using GFP-labeled macrophages to detect engulfed cells as dark spots in the cytoplasm also indicated that average numbers of engulfed live thymocytes per 100 macrophages (phagocytosis index) decreased from 18.6 to 3.2 in the presence of the αICAM-1 antibody (Fig. 3d and e). To determine which cell types can be engulfed *via* ICAM-1, we performed experiments using heterogeneous splenocytes as prey instead of thymocytes and identified phagocytosed cell types using immunocytochemical analyses of cell-specific markers. These analyses showed that the αICAM-1 antibody specifically inhibited the phagocytosis of live CD4^+^ and CD8^+^ T cells but not B cells (Fig. 3f). Blockade of ICAM-1 also inhibited the phagocytosis of live T cell lymphoma (EL4) but not other cell types such as myeloma (NSO) by stimulated macrophages (Fig. S2b). Taken together, these results suggest that the ICAM-1 receptor is induced in CpG-, IFN-γ-, and αIL-10R-cotreated macrophages and mediates the phagocytosis of live T cells.

### 3.4 Reconstitution of Live Cell Phagocytosis With NIH3T3 Cells

**Fig. 4:**
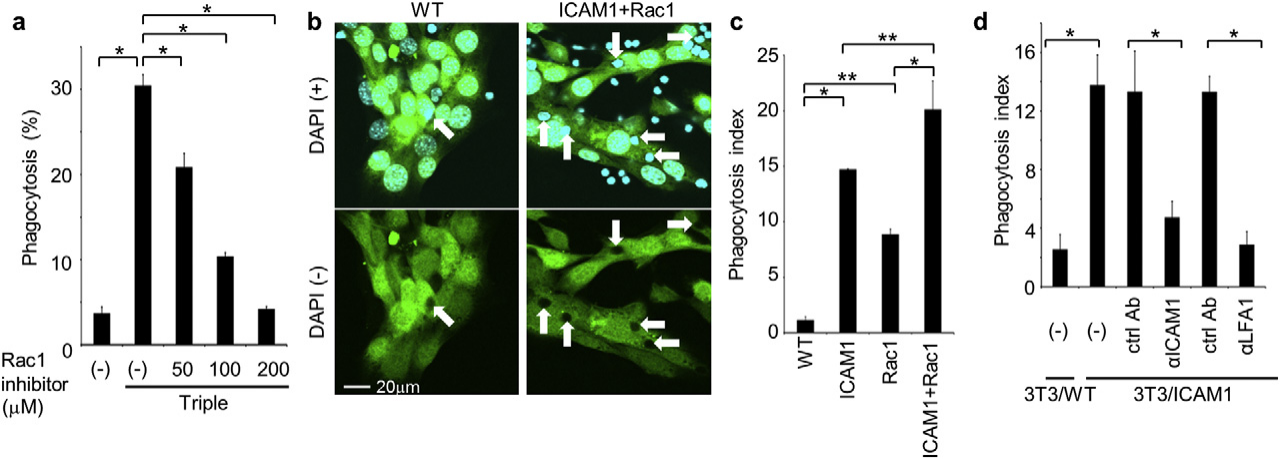
NIH3T3 cells with reconstituted live cell phagocytosis. (a) BMDMs were activated or not with CpG, IFN-γ, and αIL-10R cotreatment (Triple) in the absence (−) or presence of the Rac1 inhibitor NSC23766, and were then co-cultured with CMFDA-labeled live thymocytes. Percentages of macrophages with CMFDA-positive live thymocytes were determined using flow cytometry; *p < 0.01, ANOVA followed by Tukey *post hoc* test. (b) GFP-labeled NIH3T3 transformants (GFP-NIH3T3) expressing control vector (WT) or ICAM-1 and Rac1 vectors were co-cultured with live thymocytes, counter-stained with DAPI (blue), and then observed using confocal microscopy (upper panels). Images without DAPI staining are shown in the lower panels to identify engulfed cells as dark spots in the cytoplasm. Arrows indicate engulfed thymocytes. (c) Numbers of engulfed live thymocytes per 100 NIH3T3 cells were counted and phagocytosis indexes were calculated; *p < 0.01, **p < 0.05, ANOVA followed by Tukey *post hoc* test. (d) GFP-NIH3T3 expressing control vector (WT) or ICAM-1 were co-cultured with live thymocytes in the absence (−) or presence of 10 μg/ml αICAM-1, αLFA-1, or respective isotype ctrl Abs, and phagocytosis indexes were calculated; *p < 0.01, Student's *t*-test. (For interpretation of the references to colour in this figure legend, the reader is referred to the web version of this article.)

Because efferocytosis has been associated with activation of the small GTPase Rac1 (Nakaya et al., 2008), we considered the possibility that Rac1 also regulates live cell phagocytosis and performed experiments using a specific Rac1 inhibitor. Following costimulation with CpG, IFN-γ, and αIL-10R, live cell phagocytosis was inhibited by the Rac1 inhibitor in a dose-dependent manner (Fig. 4a). Thus, to examine the roles of ICAM-1 and Rac1 in the phagocytosis of live cells, we reconstituted live cell phagocytosis by using GFP-labeled NIH3T3 fibroblast cells, which do not normally engulf live cells. The NIH3T3 transformants showed strongly enhanced phagocytosis of co-cultured thymocytes detected as dark spots in the cytoplasm (Fig. 4b). Specifically, overexpression of ICAM-1 or Rac1 increased phagocytosis indexes from 1.1 to 14.7 or 8.9, respectively, and overexpression of both ICAM-1 and Rac1 further increased this index to 20.1 (Fig. 4c). This level was equivalent to that in stimulated macrophages (Fig. 3e), indicating that ICAM-1 and Rac1 are essential and sufficient mediators of live T cell phagocytosis. Interestingly, overexpression of Rac1 alone increased the phagocytosis to some extent probably *via* endogenous ICAM-1, because Rac1 activation did not affect ICAM-1 expression level (data not shown). ICAM-1 reportedly binds LFA-1 (Marlin and Springer, 1987), suggesting that live T cells express LFA-1 as a phagocytic ligand. Accordingly, blockade of LFA-1 using a neutralizing antibody inhibited the phagocytosis of live thymocytes by ICAM-1 expressing NIH3T3 transformants as efficiently as the αICAM-1 antibody (Fig. 4d). The cytoplasmic tail of ICAM-1 has been shown to transduce intracellular signals (Holland and Owens, 1997). Thus, in further experiments, we generated an ICAM-1 deletion mutant that lacks the cytoplasmic domain and determined phagocytic activities in NIH3T3 transformants. However, these cells engulfed live thymocytes as efficiently as those expressing full-length ICAM-1, suggesting that ICAM-1 itself does not transduce the signal for phagocytosis (Fig. S2c).

### 3.5 Both ICAM-1 and VCAM-1 are Required for Phagocytosis of Live B Cells

**Fig. 5:**
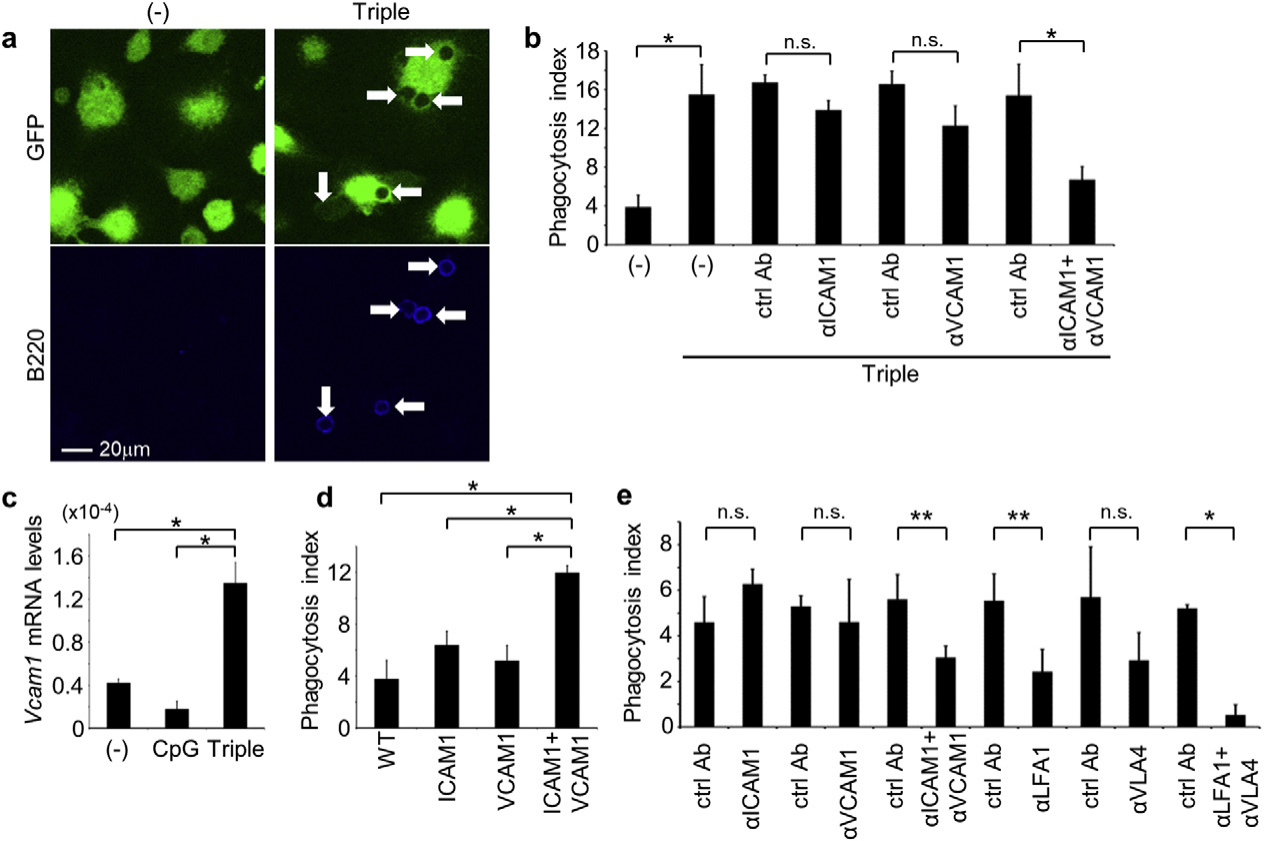
Cooperation of ICAM-1 and VCAM-1 in phagocytosis of live B cells. (a) GFP-BMDMs were activated or not (−) with CpG, IFN-γ, and αIL-10R cotreatment (Triple) and were co-cultured with live splenocytes. Cells were then fixed, permeabilized, and stained for B220 (lower panels). Engulfed cells can be detected as dark spots in the cytoplasm (upper panels). Arrows indicate engulfed B cells. (b) GFP-BMDMs activated or not with cotreatment (Triple) were co-cultured with live splenocytes in the absence (−) or presence of 4 μg/ml αICAM-1 and/or αVCAM-1, or respective isotype ctrl Abs. Numbers of engulfed B220-positive cells per 100 macrophages were determined as phagocytosis indexes; *p < 0.01; n.s., not significant, Student's *t*-test. (c) qPCR analyses were performed to compare *Vcam-1* mRNA levels in BMDMs following activation or not (−) with CpG or CpG, IFN-γ, and αIL-10R (Triple). Gene expression levels are presented relative to that of *β-actin*; *p < 0.01, ANOVA followed by Tukey *post hoc* test. (d) GFP-NIH3T3 expressing control vector (WT), ICAM-1, and/or VCAM-1 were co-cultured with live splenocytes, and numbers of engulfed B220-positive cells per 100 NIH3T3 cells were determined as phagocytosis indexes; *p < 0.01, ANOVA followed by Tukey *post hoc* test. (e) GFP-NIH3T3 expressing both ICAM-1 and VCAM-1 were co-cultured with live splenocytes in the presence of various combinations of antibodies or respective isotype ctrl Abs at 10 μg/ml, and phagocytosis indexes of B220-positive cells were determined; *p < 0.01, **p < 0.05; n.s., not significant, Student's *t*-test.

To identify mediators of live B cell phagocytosis, we used freshly-isolated splenocytes as prey and determined numbers of B220-positive cells engulfed by macrophages that had been costimulated with CpG, IFN-γ, and αIL-10R (Fig. 5a). In these experiments, the effects of blocking antibodies against candidate proteins were examined. Whereas no single antibody inhibited the phagocytosis of live B cells, the combination of αICAM-1 + αVCAM-1 antibodies did (Fig. 5b). Thus, we confirmed that *Vcam-1* mRNA levels were upregulated in the present stimulated macrophages, whereas observed no changes following stimulation with CpG alone (Fig. 5c). Furthermore, overexpression of ICAM-1 and VCAM-1 in NIH3T3 cells enhanced the phagocytosis of live B cells, increasing the phagocytosis index from 3.8 to 12.0, a level equivalent to that in stimulated macrophages, whereas overexpression of ICAM-1 or VCAM-1 individually had limited effects (Fig. 5d). As ICAM-1 and VCAM-1 bind LFA-1 and VLA-4, respectively (Marlin and Springer, 1987, Elices et al., 1990), the enhanced phagocytosis of live B cells by NIH3T3 transformants expressing both ICAM-1 and VCAM-1 was inhibited by a combination of αICAM-1 + αVCAM-1 or αLFA-1 + αVLA-4 antibodies (Fig. 5e). However, no single antibody had profound effects, indicating that ICAM-1 and VCAM-1 ligand binding and signaling play cooperative and compensatory roles in the phagocytosis of live B cells.

### 3.6 Phagocytosis of Live Myeloid Cells Depends on VCAM-1 and Integrin α_V_β_3_

**Fig. 6:**
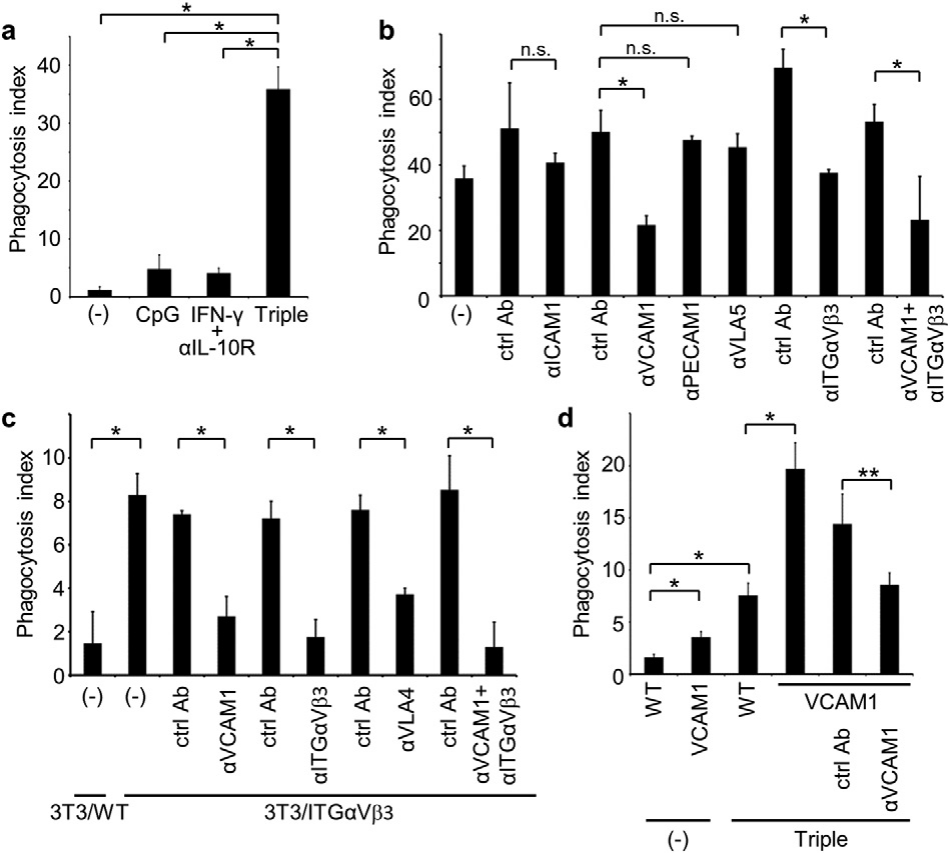
Phagocytosis of live myeloid cells *via* VCAM-1 and integrin α_V_β_3_. (a) GFP-BMDMs were stimulated or not (−) with CpG, IFN-γ + αIL-10R, or CpG, IFN-γ, and αIL-10R (Triple) and were co-cultured with freshly-isolated live myeloid cells. Numbers of engulfed live myeloid cells per 100 macrophages were determined as the phagocytosis indexes; *p < 0.01, Student's *t*-test. (b) Following activation with CpG, IFN-γ, and αIL-10R, GFP-BMDMs were co-cultured with live myeloid cells in the presence of indicated antibodies or respective isotype ctrl Abs at 4 μg/ml, and numbers of engulfed live myeloid cells per 100 macrophages were determined as the phagocytosis indexes; *p < 0.01; n.s., not significant, Student's *t*-test. (c) GFP-NIH3T3 expressing control vector (WT) or integrin (ITG) α_V_β_3_ were co-cultured with live myeloid cells in the absence (−) or presence of indicated antibodies or respective isotype ctrl Abs at 10 μg/ml, and numbers of engulfed live myeloid cells per 100 NIH3T3 cells were determined; *p < 0.01, Student's *t*-test. (d) GFP-BMDMs expressing control vector (WT) or VCAM-1 were activated or not (−) by cotreatment with CpG, IFN-γ, and αIL-10R (Triple), and were co-cultured with live myeloid cells in the absence or presence of αVCAM-1 or isotype ctrl Ab. Numbers of engulfed live myeloid cells per 100 macrophages were determined; *p < 0.01, **p < 0.05, Student's *t*-test.

We next examined whether ICAM-1 and/or VCAM-1 are involved in the phagocytosis of live cells other than lymphocytes. For this purpose, we prepared myeloid cells from bone marrow by depleting T and B cells, and confirmed that they were Annexin V negative-viable cells (Fig. S3a and b). As with live T and B cells, cotreatment of BMDMs with CpG, IFN-γ, and αIL-10R induced phagocytosis of freshly-isolated live myeloid cells, and this phagocytosis was not inhibited by the addition of the D89E mutant of MFG-E8 (Fig. 6a and data not shown). Moreover, engulfed live myeloid cells were TUNEL-negative and retained intact plasma membranes (Fig. S3c and d). To identify the mediators of this process, we examined the inhibitory effects of various neutralizing antibodies and found that αVCAM-1 and αintegrin α_V_β_3_ antibodies effectively inhibited phagocytosis of live myeloid cells, whereas other antibodies, such as αICAM-1, did not (Fig. 6b). Hence, in further experiments, NIH3T3 transformants expressing VCAM-1 and/or integrin α_V_β_3_ were established to reconstitute phagocytosis. Overexpression of integrin α_V_β_3_ significantly increased the phagocytosis of live myeloid cells, whereas overexpression of VCAM-1 did not (Fig. S3e), as reflected by sufficient expression of endogenous VCAM-1 in NIH3T3 cells (data not shown). However, enhanced phagocytosis of live myeloid cells by NIH3T3 transformants expressing integrin α_V_β_3_ was inhibited by antibodies against VCAM-1, integrin α_V_β_3_, and VLA-4, whereas antibodies against other molecules had no effect (Fig. 6c and S3f). Thus, to confirm pro-phagocytic effects, VCAM-1 was stably expressed in BMDMs, and moderate enhancements of live myeloid cell phagocytosis were observed in the absence of stimulation (Fig. 6d). In contrast, when BMDMs were stimulated with CpG, IFN-γ, and αIL-10R, overexpression of VCAM-1 further enhanced the phagocytosis of live myeloid cells, and the αVCAM-1 antibody abolished this effect. Blockade of VCAM-1 or VLA-4 also inhibited phagocytosis of live NSO myeloma cells by stimulated macrophages and NIH3T3 transformants expressing integrin α_V_β_3_ (Fig. S3g and h). Taken together, these results indicate that phagocytosis of live myeloid cells is mediated by VCAM-1 and integrin α_V_β_3_.

### 3.7 Live Cell Phagocytosis by Other Phagocytes and *In Vivo* Model

**Fig. 7:**
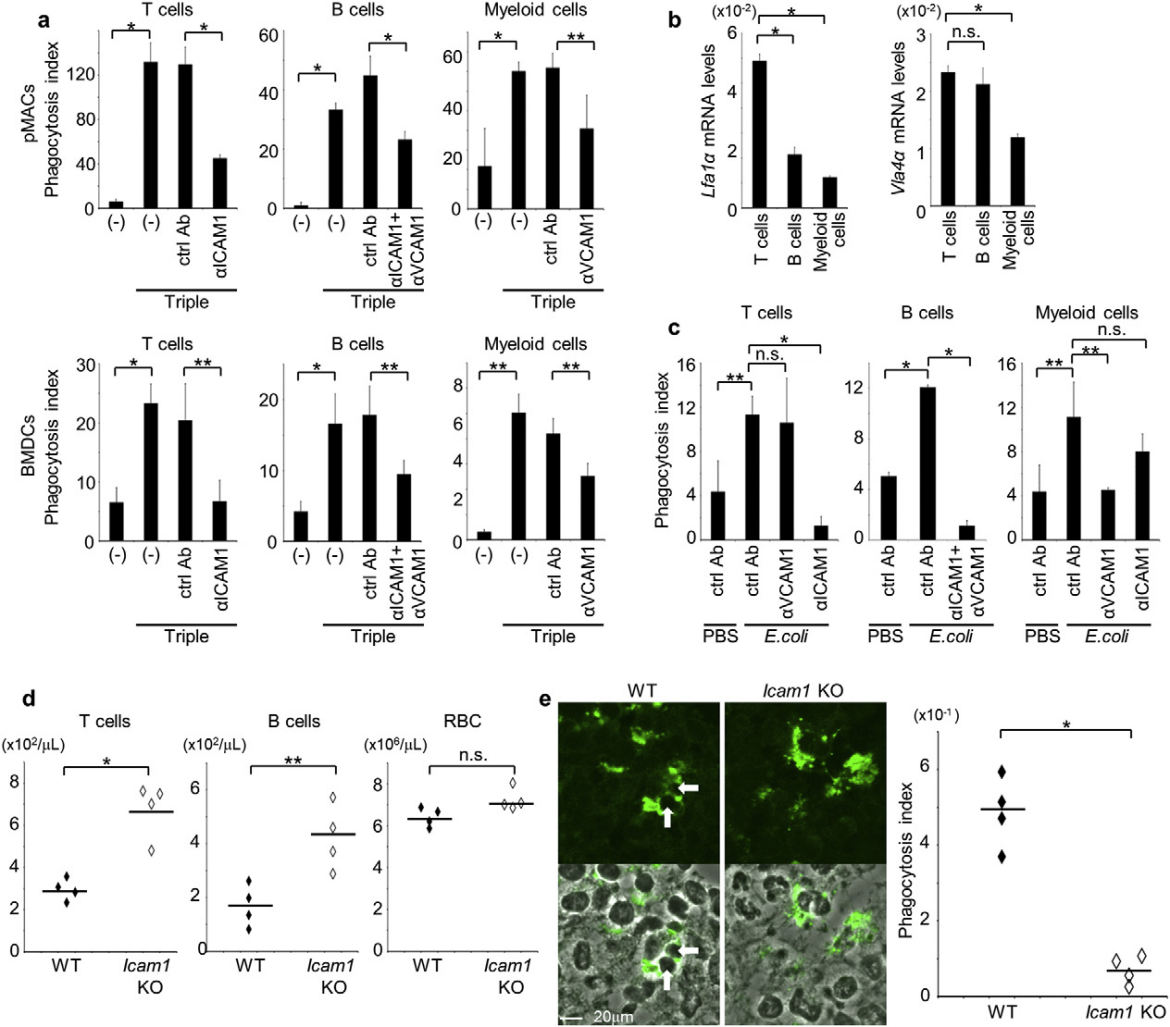
ICAM-1 and VCAM-1 mediate live cell phagocytosis *in vivo*. (a) GFP-labeled pMACs (upper graphs) or BMDCs (lower graphs) were cotreated or not with CpG, IFN-γ, and αIL-10R (Triple) and were co-cultured with live thymocytes, splenocytes, or myeloid cells in the absence (−) or presence of αICAM-1 and/or αVCAM-1, or respective isotype ctrl Abs at 10 μg/ml. Numbers of engulfed live cells per 100 phagocytes were determined as the phagocytosis indexes; *p < 0.01, **p < 0.05, Student's *t*-test. (b) qPCR analyses were performed to compare *Lfa-1α* and *Vla-4α* mRNA levels between T, B, and myeloid cells. T and B cells were collected from splenocytes using a FACSAria cell sorter with αCD3 and αB220 antibodies. Gene expression levels are presented relative to that of *β-actin*; *p < 0.01; n.s., not significant, Student's *t*-test. (c) Mice received intraperitoneal injections of PBS or dead *E. coli* (200 μg). After 2 days, the mice were injected with CMFDA-labeled freshly-isolated live thymocytes, splenocytes, or myeloid cells into the peritoneal cavities in the presence of indicated antibodies or respective isotype ctrl Abs (10 μg). After 2.5 h, total numbers of engulfed live cells in 100 CD11b^+^/Gr-1^−^ peritoneal macrophages were determined as phagocytosis indexes. Experiments were performed 3 times independently, and average values were plotted with s.d.; *p < 0.01, **p < 0.05; n.s., not significant, Student's *t*-test. (d) WT or *Icam-1* KO mice were treated over 8-day period with repeated intraperitoneal injections of dead *E. coli* (20 mg) every 2 days. On day 7, peripheral blood was sampled, and each blood cell count was examined by a flow cytometry. Experiments were performed 4 times independently. Small horizontal lines indicate the mean; *p < 0.01, **p < 0.05; n.s., not significant, Student's *t*-test. (e) After repeated dead *E. coli* injections, spleens from WT or *Icam-1* KO mice were taken on day 10, and were analyzed by immunohistochemistry with αCD68 antibody (green). Staining profiles were merged with the phase contrast images in the lower panels. Arrows indicate engulfed live cells. Numbers of engulfed cells per 100 CD68^+^ macrophages were determined as phagocytosis indexes and plotted in the right graph; *p < 0.01, Student's *t*-test. (For interpretation of the references to colour in this figure legend, the reader is referred to the web version of this article.)

To determine whether phagocytes other than BMDMs are subject to the present mechanisms of live cell phagocytosis, we co-cultured pMACs or BMDCs with live thymocytes, splenocytes, or myeloid cells. Under these conditions, phagocytosis was initiated by cotreatment with CpG, IFN-γ, and αIL-10R in pMACs and in BMDCs (Fig. 7a). Furthermore, phagocytosis of live T cells, B cells, or myeloid cells was specifically inhibited by αICAM-1, αICAM-1 + αVCAM-1, or αVCAM-1 antibodies, respectively, confirming that phagocytosis is performed with cell-type specific receptors. Thus, to investigate functional consequences of these distinctions, we examined gene expression of α subunits of LFA-1 and VLA-4 in live target cells (Fig. 7b). Expression levels of *Lfa-1α* and *Vla-4α* mRNA were lowest in myeloid cells, and *Lfa-1α* mRNA levels were about 5- and 2-fold higher in T cells and B cells, respectively. Similarly, *Vla-4α* mRNA levels were about 2-fold higher in T and B cells. Hence, receptors for live cell phagocytosis correlate with LFA-1 expression levels in target cells, indicating that phagocytes use ICAM-1 to recognize cells with high LFA-1 expression, and that VCAM-1 is increasingly used with decreasing LFA-1 expression in target cells. Finally, we determined the effects of ICAM-1 and VCAM-1 in *in vivo* models of inflammation. In these experiments, injections of non-viable *E.coli* into mouse peritoneal cavities led to significant increases in *Icam-1* and *Vcam-1* mRNA levels in CD11b^+^/Gr-1^−^ peritoneal macrophages after 2 days (Fig. S4a and b). Following injection into peritoneal cavities, freshly-isolated live cells were efficiently engulfed by peritoneal macrophages that had been stimulated with non-viable *E.coli* (Fig. 7c). However, subsequent co-injections of αICAM-1, αICAM-1 + αVCAM-1, and αVCAM-1 specifically inhibited the phagocytosis of live T cells, B cells, and myeloid cells, respectively. These results are consistent with the present *in vitro* experiments, indicating that ICAM-1 and VCAM-1 play pivotal roles in cell-type specific live cell phagocytosis. To further determine the involvement of ICAM-1 in an *in vivo* model, we generated *Icam-1* KO mice. Repeated administration of non-viable *E.coli* to WT mice resulted in a peripheral pancytopenia (Fig. S4c), but reductions in T and B cell numbers was ameliorated in *Icam-1* KO mice (Fig. 7d). Accordingly, while WT mice developed hemophagocytosis in spleens, it was rarely found in the *Icam-1* KO mice (Fig. 7e). These results further indicate that ICAM-1 mediates live cell phagocytosis *in vivo*.

## 4 Discussion

**Fig. 8:**
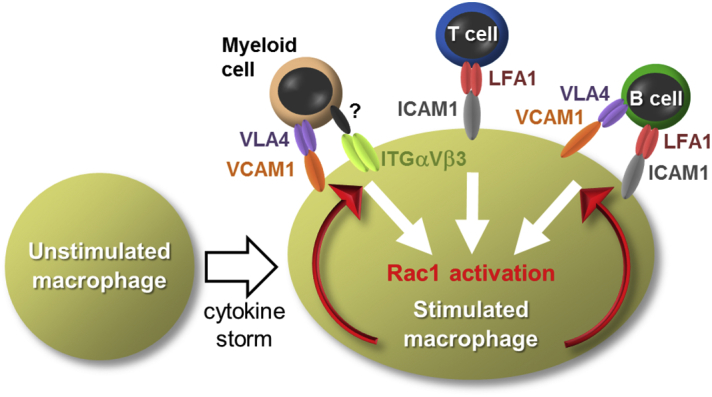
Schematic model of live cell phagocytosis by macrophages. Cytokine storm induces up-regulation of cell-type specific receptors on the stimulated macrophages for tethering of living cells. VCAM-1, ICAM-1, or ICAM-1/VCAM-1 mediates tethering of myeloid cells, T, or B lymphocytes, respectively to the macrophages. Integrin α_V_β_3_ might work as co-receptors which transduce a phagocytic signaling for Rac1 activation to promote the internalization of the bound cells.

In this study, we found ICAM-1 and VCAM-1 were induced in macrophages by a combination of inflammatory stimuli and they subsequently played important roles in live cell phagocytosis (Fig. 8). ICAM-1 and VCAM-1 have been identified as ligands for LFA-1 and VLA-4, respectively, and their functions in the control of migration, adhesion, and activation of lymphocytes are well established (Marlin and Springer, 1987, Elices et al., 1990). The present data further clarified functions as receptors for live cell phagocytosis. Specifically, reconstitution experiments in NIH3T3 cells showed that intracellular regions of ICAM-1 are dispensable for transducing phagocytic signaling. Therefore, these proteins likely tether target live cells to macrophages prior to phagocytosis. Consistently, we previously proposed a two-step efferocytosis model in which apoptotic cells were tethered to macrophages prior to engulfment (Hanayama et al., 2004, Toda et al., 2012). In this model, macrophage phosphatidylserine-binding proteins, such as Tim4 and MFG-E8, recognize phosphatidylserine on apoptotic cells. However, as is seen with ICAM-1, the intracellular region of Tim4 is dispensable for transducing phagocytic signaling, indicating that Tim4 tethers apoptotic cells to macrophages. In contrast, MFG-E8 mediates engulfment of apoptotic cells as a ligand for integrin α_V_β_3_, which activates Rac1. Application of this two-step model to live cell phagocytosis suggests that ICAM-1 and VCAM-1 tether live cells to macrophages in response to inflammatory stimuli, but other receptors are required for subsequent engulfment. The reported roles of integrin α_V_β_3_ as a receptor for phagocytosis of live myeloid cells support this idea, but its ligand remains unidentified. Moreover, blockade of integrin α_V_β_3_ had only minimal effects on the phagocytosis of live T and B cells (data not shown), indicating the presence of other integrins or integrin-like proteins that mediate live cell phagocytosis. Rac1 is reportedly activated following stimulation of ligands with integrins, and facilitates actin-remodeling for the engulfment (Albert et al., 2000). However, under the conditions of a cytokine storm, IL-1 and IFN-γ can activate Rac1 in the absence of integrins (Windheim and Hansen, 2014, Frausto-Del-Rio et al., 2012). Therefore, tethering alone may be sufficient for the phagocytosis of live cells under conditions of severe inflammation.

Strong expression of ICAM-1 and VCAM-1 which cause live cell phagocytosis cannot be induced by the individual stimulation, indicating that crosstalk between the inflammatory signaling pathways is essential for this phenomenon. Indeed, TLR signaling is known to amplify IFN-γ signaling through phosphorylation of STAT1 on serine 727 *via* p38 (Dalpke et al., 2003). It is also known that p38 activated by CpG-stimulated TLR signaling induce IL-10 production, which in turn suppress both TLR and IFN signaling pathways (Yi et al., 2002). By blocking the negative feedback effect, the addition of αIL-10R enhanced the live cell phagocytosis which was induced by a combination of CpG and IFN-γ to some extent (data not shown).

Phagocytosis of erythrocytes and platelets plays important pathophysiological roles in HLH (Janka, 2012, Janka and Lehmberg, 2014), and severe reductions of platelet numbers causes fatal conditions such as cerebral hemorrhage and gastrointestinal bleeding. In the present experiments, although treatments with CpG, IFN-γ, and αIL-10R promoted phagocytosis of live erythrocytes and platelets (data not shown), we were unable to identify the induced molecules that are responsible for phagocytosis of live erythrocytes and platelets, warranting further studies using this cell culture model. Although erythrocytes and platelets reportedly expose phosphatidylserine readily during aging and activation (Nagata et al., 2016), phagocytosis occurred independently of phosphatidylserine in our experiments (data not shown).

As the regulatory mechanisms of efferocytosis become clear, physiological functions in the maintenance of tissue homeostasis and prevention of autoimmune disease and chronic inflammation are implied (Nagata et al., 2010, Arandjelovic and Ravichandran, 2015). Although the physiological functions of hemophagocytosis have not been clarified, it may be a homeostatic mechanism to facilitate resolution of excessive inflammation by depleting immune cells. Live cell phagocytosis or cell-in-cell structures, in which live cells are internalized into other cells, can be observed in various situations besides hemophagocytosis (Overholtzer and Brugge, 2008). One such mechanism is leukocyte transcellular migration, which is dependent on leukocyte's interaction with several receptors on endothelial cells, including ICAM-1, VCAM-1, or PECAM-1 (Muller, 2011). Another example is thymic nurse cell internalization of thymocytes: the epithelial nurse cells in the cortex of the thymus reportedly internalize up to 50 live thymocytes to provide microenvironments that optimize T-cell selection (Nakagawa et al., 2012). Entosis is a homotypic cell-in-cell structure triggered by loss of attachment to the extracellular matrix (Overholtzer et al., 2007). The invasion of one cell into another is driven by compaction force that is activated by the small GTPase Rho and its effector kinase Rho-kinase (ROCK). In any case, the internalized cells remain alive inside their hosts for extended periods, and can occasionally undergo cell division or escape. The present data indicate that delayed P–L fusion inside the host cells may contribute to the survival of the internalized cells. On the other hand, cell death executed by phagocytosis of live cells is known as phagoptosis or primary phagocytosis (Brown and Neher, 2012). Phagocytosis of an otherwise-viable cell may occur as a result of cell stress, activation or senescence, which is involved in turnover of erythrocytes and other cells. Phagoptosis is usually caused by molecular machinery similar to efferocytosis such as phosphatidylserine exposure, which is different from live cell phagocytosis established in our current model.

Studies of hemophagocytosis have mainly been performed using *in vivo* models or human studies, which are not sufficient for comprehensive analyses of molecular mechanisms. Given the consistency of the present *in vitro* cell culture assay, this model will likely contribute to understanding of the molecular mechanisms of hemophagocytosis, and will facilitate the development of therapeutic approaches against it.

## Funding Sources

This research was supported by grants from JST PRESTO “Chronic Inflammation” (No. 4336 to R.H.).

## Appendix A Supplementary data

Supplementary figures Image 1
