# Supplementary material for: Induction of Live Cell Phagocytosis by a Specific Combination of Inflammatory Stimuli
Source: eBioMedicine. 2017 Jul 13;22:89–99. doi: 10.1016/j.ebiom.2017.07.011 (PMC5552246; doi:10.1016/j.ebiom.2017.07.011)
Supplement: Supplementary file 1 — Supplementary figures [file mmc1.docx]

**Supplemental material, Ishidome et al.**

**Supplemental Figures**


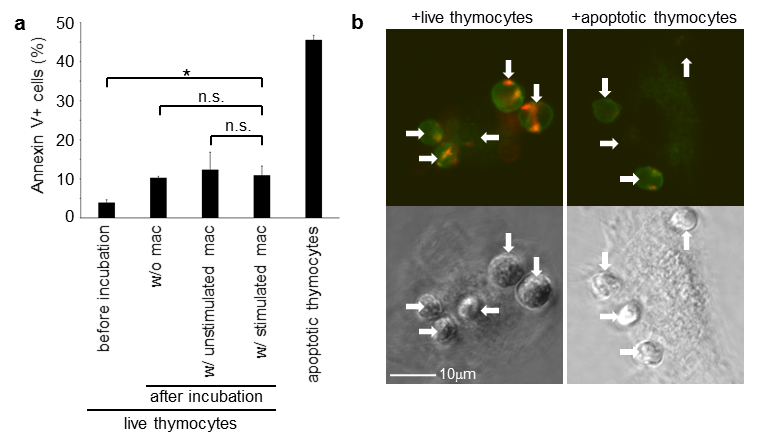


**Fig. S1.** Engulfment of live cells. (a) Freshly-isolated live thymocytes were stained for Annexin V before or after incubation with or without BMDMs (mac), and the percentage of Annexin V-positive cells were determined by flow cytometry. Apoptotic thymocytes were used as a positive control. Experiments were performed in triplicates, and average values were plotted with s.d.; **p* < 0.05; n.s., not significant, Student’s *t*-test. (b) Live or apoptotic thymocytes engulfed by BMDMs were stained by JC-1 assay and observed using confocal microscopy (upper panels). Phase contrast images of the cells are shown in the lower panels. Arrows indicate engulfed live thymocytes. Live cells maintaining mitochondrial membrane potential emit red signals.


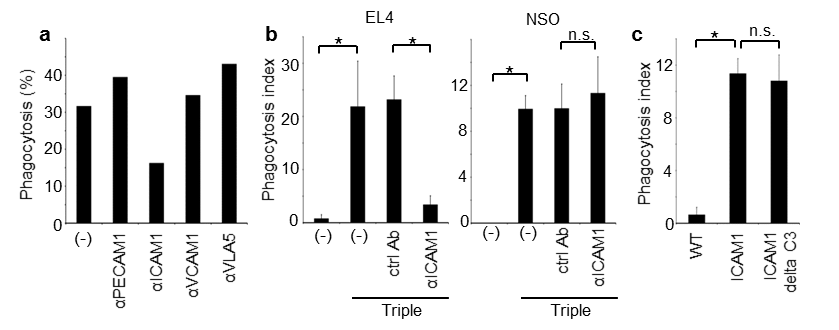


**Fig. S2.** ICAM-1 mediates phagocytosis of live T cells. (a) BMDMs were activated by cotreatment with CpG, IFN-γ, and αIL-10R and were then co-cultured with CMFDA-labeled live thymocytes in the absence (−) or presence of the blocking antibodies αPECAM-1, αICAM-1, αVCAM-1, or αVLA-5 at 4 μg/ml. Percentages of CMFDA-positive macrophages were quantified using flow cytometry. (b) GFP-BMDMs were cotreated or not with CpG, IFN-γ, and αIL-10R (Triple) and were then co-cultured with live EL4 or NSO cells in the absence (−) or presence of αICAM-1 or isotype ctrl Ab at 4 μg/ml. Numbers of engulfed live cells per 100 macrophages were determined as phagocytosis indexes, and average values were plotted with s.d.; **p* < 0.01; n.s., not significant, Student’s *t*-test. (c) GFP-NIH3T3 expressing control vector (WT), full length ICAM-1, or ICAM-1 lacking the cytoplasmic domain (ICAM-1 delta C3) were co-cultured with live thymocytes, and phagocytosis indexes were determined; **p* < 0.01; n.s., not significant, Student’s *t*-test.


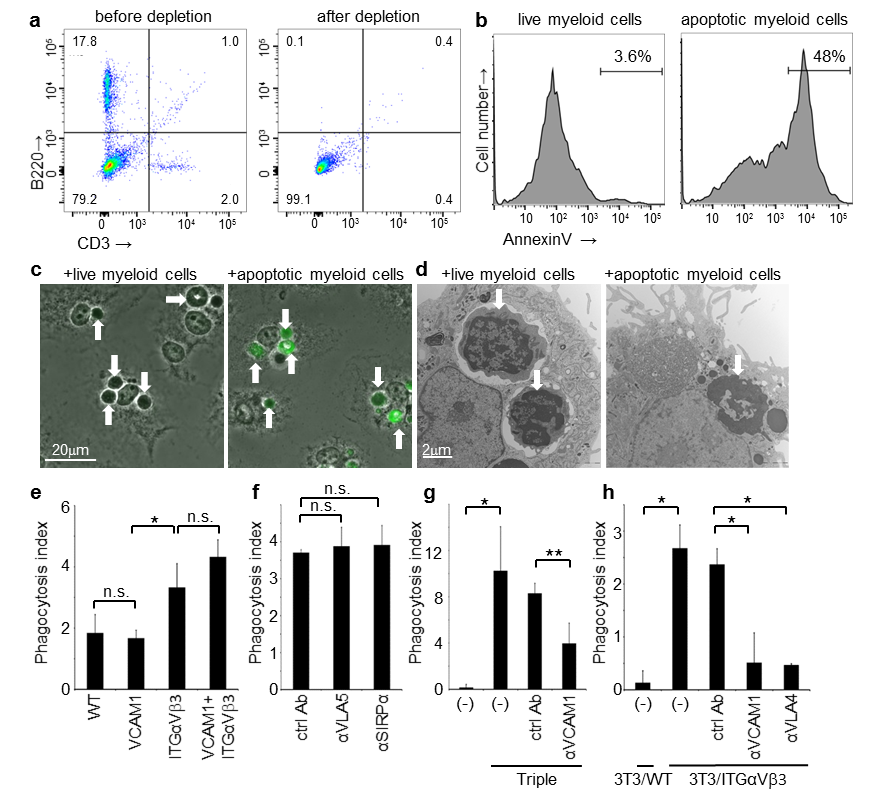


**Fig. S3.** VCAM-1 mediates phagocytosis of live myeloid cells. (a) Freshly-isolated bone marrow cells were stained for B220 and CD3 (before depletion), and B220^+^ cells and CD3^+^ cells were depleted by using a FACSAria cell sorter (after depletion). (b) After cell sorting, the live myeloid cells or apoptosis-induced myeloid cells were stained with Annexin V, and % of Annexin V-positive cells was quantified by flow cytometry. (c and d) BMDMs were activated by cotreatment with CpG, IFN-γ, and αIL-10R, co-cultured with live or apoptotic myeloid cells, and then analyzed using TUNEL staining (green) (c) or electron microscopy (d). Apoptosis of myeloid cells was induced by UV irradiation at 200 J. Arrows indicate myeloid cells that were engulfed by macrophages. (e) GFP-NIH3T3 expressing control vector (WT), VCAM-1, and/or integrin (ITG) α_V_β_3_ were co-cultured with live myeloid cells, and phagocytosis indexes were determined; **p* < 0.01; n.s., not significant, Student’s *t*-test. (f) GFP-NIH3T3 expressing integrin α_V_β_3_ were co-cultured with live myeloid cells in the presence of indicated blocking antibodies or isotype ctrl Ab at 10 μg/ml, and phagocytosis indexes were determined; n.s., not significant, Student’s *t*-test. (g) GFP-BMDMs were cotreated or not with CpG, IFN-γ, and αIL-10R (Triple) and were then co-cultured with live NSO cells in the absence (−) or presence of αVCAM-1 or isotype ctrl Ab at 4 μg/ml, and phagocytosis indexes were determined; **p* < 0.01, ***p* < 0.05, Student’s *t*-test. (h) GFP-NIH3T3 expressing control vector (WT) or integrin (ITG) α_V_β_3_ were co-cultured with live NSO cells in the absence (−) or presence of αVCAM-1, αVLA-4, or respective isotype ctrl Abs at 10 μg/ml, and phagocytosis indexes were determined; **p* < 0.01, Student’s *t*-test.


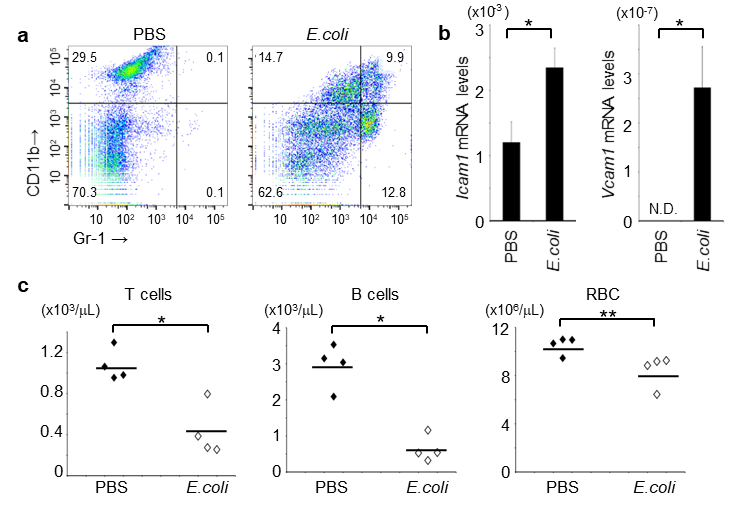


**Fig. S4.** Induction of *in vivo* live cell phagocytosis. (a) PBS or dead *E. coli* (200 μg) were injected into peritoneal cavities of mice. After 2 days, peritoneal cells were collected and stained for CD11b and Gr-1. (b) CD11b^+^/Gr-1^−^ peritoneal macrophages were collected using a FACSAria cell sorter. Total RNA was then extracted and qPCR analyses were performed to compare *Icam-1* and *Vcam-1* mRNA levels between PBS and *E.coli* exposed macrophages. Gene expression levels are presented relative to that of *β-actin*; **p* < 0.01, Student’s *t*-test. N.D., not detectable. (c) Mice received repeated intraperitoneal injections of PBS or dead *E. coli* (20 mg) for 5 times every 2 days. After the 4^th^ injection, peripheral blood was sampled and each blood cell count was examined by a flow cytometry. Experiments were performed 4 times independently. Small horizontal lines indicate the mean; **p* < 0.01, ***p* < 0.05, Student’s *t*-test.
